# CCN5 Drives Leydig Cell Aging and Testicular Dysfunction: Insights into Fibrosis, Lipid Dysregulation, and Therapeutic Potential

**DOI:** 10.34133/research.0762

**Published:** 2025-08-01

**Authors:** Xiaoli Tan, Yanghua Xu, Ningjing Ou, Yuzhuo Chen, Wanyi Xia, Emily Xing, Ren Mo, Hao Bo, Zhitao Han, Jiarong Xu, Zepeng Dong, Yingbo Dai, Yuxin Tang, Liangyu Zhao

**Affiliations:** ^1^ Department of Urology, The Fifth Affiliated Hospital, Sun Yat-sen University, Zhuhai, Guangdong, China.; ^2^ Department of Traditional Chinese Medicine, The Fifth Affiliated Hospital, University of Sun Yat-sen, Zhuhai, China.; ^3^ Cedars-Sinai Medical Center, Los Angeles, CA, USA.; ^4^ Department of Urology, Inner Mongolia People’s Hospital, Inner Mongolia Urological Institute, Hohhot, Inner Mongolia 010017, China.; ^5^ NHC Key Laboratory of Human Stem Cell and Reproductive Engineering, Institute of Reproductive and Stem Cell Engineering, School of Basic Medical Science, Central South University, Changsha, Hunan, China.; ^6^ Clinical Research Center for Reproduction and Genetics in Hunan Province, Reproductive and Genetic Hospital of CITIC-Xiangya, Changsha, Hunan, China.

## Abstract

Leydig cells’ (LCs’) senescence is an important reason for the decline of testicular function in elderly men. Cellular communication network factor 5 (CCN5) regulates lipid metabolism and cellular fibrosis through multiple mechanisms. However, its role in LCs’ aging and the underlying molecular mechanisms remain unclear. This study aimed to elucidate the effects and molecular mechanisms by which CCN5 drives aging phenotypes in LCs and to evaluate the potential of targeting CCN5 as a therapeutic strategy for testicular aging. CCN5 expression was located in LCs and elevated in aged testis. Overexpression of CCN5 led to LCs’ aging and testis dysfunction. Extracellularly, CCN5 activated β-catenin and SMAD2/3 phosphorylation, promoting the expression of fibrosis-related genes. Intracellularly, CCN5 did not affect de novo cholesterol synthesis-related genes but changed the balance of cholesterol transporters. CCN5 bound to and reduced ring finger protein 213 (RNF213) protein levels. RNF213 knockdown activated forkhead box O, p16, and p21, resulting in SA-β-gal activation, reduced cell proliferation, and lipid droplet loss. In aged mice, CCN5 knockdown improved testicular atrophy, restored lipid droplet content and testosterone synthesis, and enhanced physical endurance and sexual behavior. In summary, CCN5 drives LCs’ aging and testicular dysfunction maybe via promoting fibrosis and lipid droplet loss. Targeting CCN5 offers a promising strategy to treat testicular aging and associated reproductive endocrine disorders.

## Introduction

Maintaining spermatogenesis and reproductive endocrine homeostasis are the 2 most essential functions of the testis. Testicular aging not only affects male fertility but also leads to late-onset hypogonadism (LOH), characterized by a decline in serum testosterone levels and symptoms such as osteoporosis, muscle atrophy, fatigue, memory loss, sleep disturbances, and sexual dysfunction [1]. Morphological changes in aging testes include reduced testicular weight and density, decrease in germ cells and Sertoli cells, thickened basement membranes of seminiferous tubules, and interstitial fibrosis [2,3]. Notably, these features also appear in some pathological conditions, suggesting the presence of premature aging phenotypes in testis. For example, idiopathic non-obstructive azoospermia (iNOA) is characterized by thickened and fibrotic basement membranes of seminiferous tubules, altered collagen composition, increased senescence-associated secretory phenotype proteins, and reduced serum testosterone levels [4]. Similarly, patients with Klinefelter’s syndrome exhibit seminiferous tubule atrophy, interstitial hyperplasia, and fibrosis after puberty [5]. Therefore, a deeper understanding of the progression and regulatory mechanisms of testicular aging could aid in discovering methods to delay testicular functional decline, and also helpful for providing novel insights into the treatment of testis-related diseases.

Leydig cells (LCs) utilize cholesterol as a substrate to produce over 90% of the body’s testosterone. In aging LCs, intracellular lipid droplet loss signifies a deficiency in testosterone synthesis precursors, compounded by decreased expression of testosterone-synthesizing enzymes and reduced responsiveness to luteinizing hormone (LH), ultimately resulting in diminished testosterone production and secretion [6,7]. Current evidence suggests that the cholesterol in LCs is not solely derived from de novo synthesis, and cholesterol uptake defects caused by the cholesterol transporters disorder and autophagy impairment significantly reduce lipid droplet content within LCs [8]. Additionally, interstitial fibrosis in the testicular microenvironment is another important phenotype of LCs’ aging, but the regulation mechanism is still unknown.

We integrated single-cell RNA sequencing (scRNA-seq) data from multiple datasets, constructed a map of the development and aging pathways of human LCs over a large age span, and identified that cellular communication network factor 5 (CCN5) is closely related to the aging of LCs [9–13]. CCN5 is a member of the WNT1-induced secreted protein (WISP) subfamily within the connective tissue growth factor family. CCN5 protein is enriched in tissues such as the testis, ovary, myocardium, and adipose tissue; within testis, CCN5 is specifically expressed in LCs. Previous foundational research on CCN5 has predominantly focused on its roles in fibrosis and lipid metabolism regulation. In cancer models such as breast and pancreatic carcinoma, CCN5 exerts its biological effects through autocrine or paracrine mechanisms to modulate WNT signaling and other pathways, particularly influencing the endothelial–mesenchymal transition process [14–16]. This mechanism enhances tumor cell extracellular matrix (ECM) production, exacerbating cellular fibrosis. Tumor cells acquiring mesenchymal phenotypes consequently develop enhanced migratory capacity and motility, facilitating metastatic dissemination and distant colonization. In addition, CCN5 is known to activate the differentiation of mesenchymal cells into myofibroblasts and is involved in ECM remodeling, potentially explaining the fibrotic phenotypes observed in aging LCs [17–19]. Moreover, CCN5 is implicated in regulating adipocyte lineage fate, as CCN5-knockout mice exhibit lipotoxic cardiomyopathy accompanied by mild obesity and diabetes, potentially linking CCN5 to lipid droplet degradation in aging LCs [20,21]. Notably, CCN5-mediated WNT pathway regulation and its cross-talk with other signaling networks represent key areas requiring further mechanistic investigation [19,22].

Thus, we hypothesize that CCN5 serves as a key driver of LCs’ aging and its associated phenotypes. In this study, we first validated CCN5 as an effective biomarker for testicular aging using both in vivo and in vitro models. Mechanistic investigations revealed that CCN5 mediates LC fibrosis and lipid droplet loss through extracellular and intracellular pathways, respectively. Finally, by down-regulating CCN5 expression in testes, we successfully restored testicular morphology and endocrine function in aged mice, highlighting CCN5 as a promising therapeutic target for combating testicular aging.

## Results

### Lipid droplet degradation and fibrosis in LCs as key features of testicular aging

**Fig. 1.**
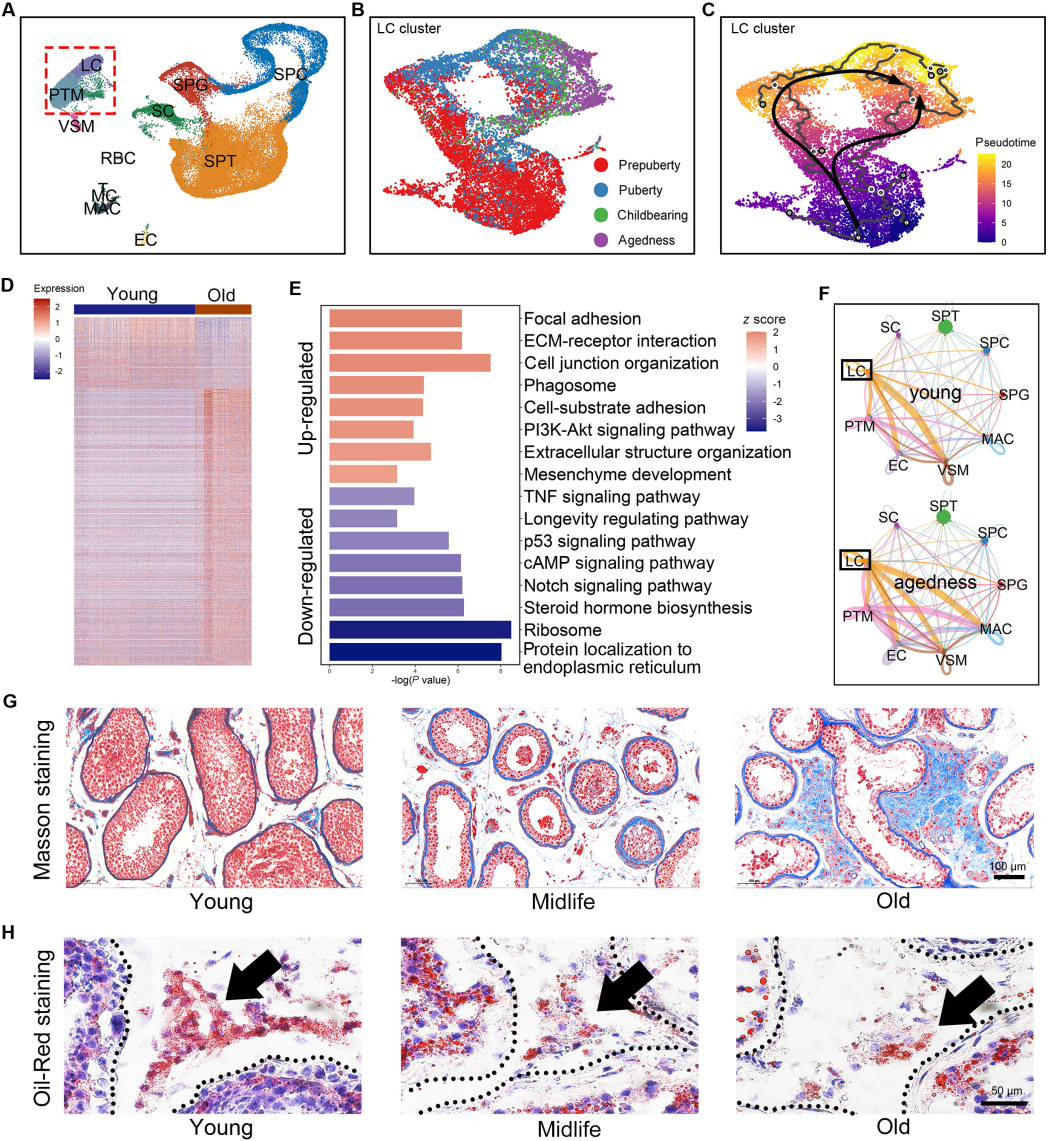
The overall characteristics of testicular microenvironmental aging. (A) Uniform manifold approximation and projection (UMAP) plots of all testis cells from different age groups. Cells are colored according to their cell types. (B and C) UMAP plot of LC cluster. Cells are colored according to their (B) age group or (C) pseudotime. (D) Heatmap of the DEGs between LCs from the young and old group. (E) GSEA terms are shown as bar plot. The *P* value is presented on the *x*-axis, and the gradient from dark blue to orange indicates low to high GSEA score. (F) Circle plots showing the cell–cell communication network within the young (top panel) and old (bottom panel) testis microenvironment. The thickness of the line represents the signal strength, and the color of the line represents the resource cell of the signal. (G) Masson's staining of human testis tissue paraffin sections from different age groups. The scale bar represents 100 μm. (H) Red oil staining of human testis tissue paraffin sections from different age groups. The arrow points to the testicular interstitial area where LCs are located. The scale bar represents 50 μm.

Aging in the testicular microenvironment show significant cellular heterogeneity, with different cell types undergoing asynchronous aging processes and exhibiting distinct senescence characteristics [10]. While the primary initiating factors of testicular aging remain unclear, the LC senescence as the main source of testosterone warrants particular attention. Therefore, we first integrated multiple scRNA-seq datasets of the testicular microenvironment, constructing a comprehensive aging trajectory map of testicular cells across the male lifespan (0 to 80 years) (Fig. 1A) [10,12,13,23]. Focusing on the LC cluster, we observed that the age of the donor closely aligned with pseudotime analysis outcomes, and analyzed the correlation between all LCs expressed genes and their pseudotime scores (Fig. 1B and C and Table S1). Comparing LCs from elderly individuals with those from healthy young adults (18 to 45 years), we identified 533 up-regulated and 249 down-regulated aging-associated genes **(**Fig. 1D and Table S2). Functional enrichment analysis of these differentially expressed genes (DEGs) revealed positive enrichment in terms such as “focal adhesion”, “extracellular structure organization”, and “mesenchyme development”, while pathways related to “Steroid hormone biosynthesis” and “Longevity regulating pathway” were negatively enriched (Fig. 1E). Additionally, ligand–receptor interaction analysis between cells indicated an enhanced interaction between LCs and macrophages (MAC) in the aged testis, suggesting an age-related inflammatory response (Fig. 1F). Previous studies have shown that aging is associated with chronic inflammation, with tissue fibrosis as a common consequence [24]. We observed that with aging, Masson staining signals in the testicular interstitial region intensified, accompanied by a local up-regulation of smooth muscle proteins such as ACTA2 and MYH11 (Fig. 1G and Fig. S1A). Additionally, tissue fibrosis is always accompanied by ECM remodeling. We also found significant alterations in the expression patterns of related proteins in elderly individuals, such as reduced levels of matrix metallopeptidase (MMP) 2/3 and laminin subunit beta 3 (LAMB3), along with the accumulation of FBLN (Fig. S1B). This phenomenon is commonly observed in various types of tissue fibrosis [25–28]. Furthermore, Oil-Red-O staining demonstrated a decrease in lipid droplet content in LCs with aging, potentially leading to a scarcity of steroidogenesis precursors (Fig. 1H) [13]. In summary, we found that intracellular lipid droplet reduction and fibrosis are important markers of LCs’ aging.

### High expression of CCN5 as an aging marker of LC

**Fig. 2.**
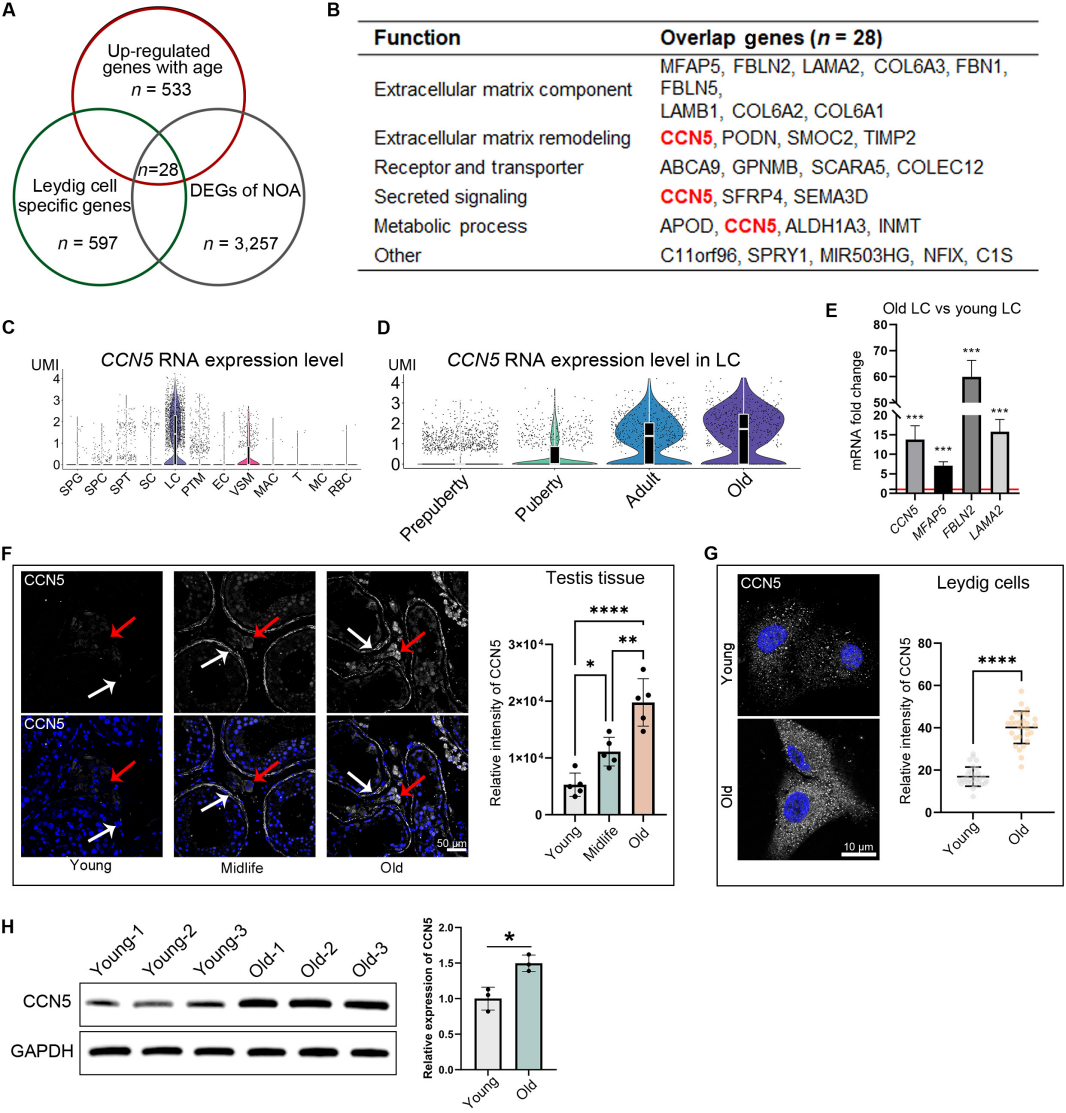
CCN5 was significantly up-regulated in aged LCs. (A) The Venn diagram shows the intersection of 3 gene sets. (B) This table shows the functional classification of these intersecting genes. (C and D) The violin plot shows the expression of CCN5 across different types of testicular cells (C), or in LCs from different age groups (D). (E) qPCR results showing the expression fold change of some ECM genes in young and old LC. *n* = 3 technical repetitions. (F) Immunofluorescence staining of CCN5 in testicular paraffin sections from different age groups. Red and white arrows mark the testis interstitial and peritubular regions of the seminiferous tubules, respectively. The scale bar represents 50 μm. The right panel shows the fluorescence intensity statistics. *n* = 5 regions. (G) Immunofluorescence of CCN5 in cultured LCs from young and old testis. The scale bar represents 10 μm. *n* = 30 cells. (H) Western blot of CCN5 protein of LCs from young and old testis.

In addition to natural aging, literature reports indicate that LCs’ aging also occurs in diseases such as non-obstructive azoospermia (NOA) and may serve as a marker for predicting testicular function [4]. To identify specific drivers of LCs’ aging, we intersected genes up-regulated in senescent LC, genes highly expressed in LCs (compared to other testicular cells), and genes up-regulated in iNOA, resulting in 28 intersecting genes (Fig. 2A). Based on LCs’ aging characteristics, we classified these genes functionally and found that CCN5 was the only gene reported to be associated with both ECM remodeling and metabolic processes (Fig. 2B). scRNA-seq data indicated that CCN5 is primarily expressed in LCs and increases significantly with age (Fig. 2C and D). To validate the sequencing results, we used qPCR to analyze *CCN5* along with several typical fibrosis-related genes, including *Microfibril Associated Protein 5* (*MFAP5*), *Fibulin 2* (*FBLN2*), and *Laminin Subunit Alpha 2* (*LAMA2*). Results showed that all these genes were up-regulated in LCs derived from elderly individuals (Fig. 2E). Furthermore, immunofluorescence staining revealed that CCN5 protein levels also increase with age in testicular tissue, especially in the interstitium and peritubular regions (Fig. 2F). Additionally, we also found higher CCN5 protein expression in LCs isolated from old individuals. These results suggest that CCN5 expression in LCs is closely linked to aging and may be a potential biomarker for assessing the biological age of the testis.

### Overexpression of CCN5 induces LCs’ aging in vivo and in vitro

**Fig. 3.**
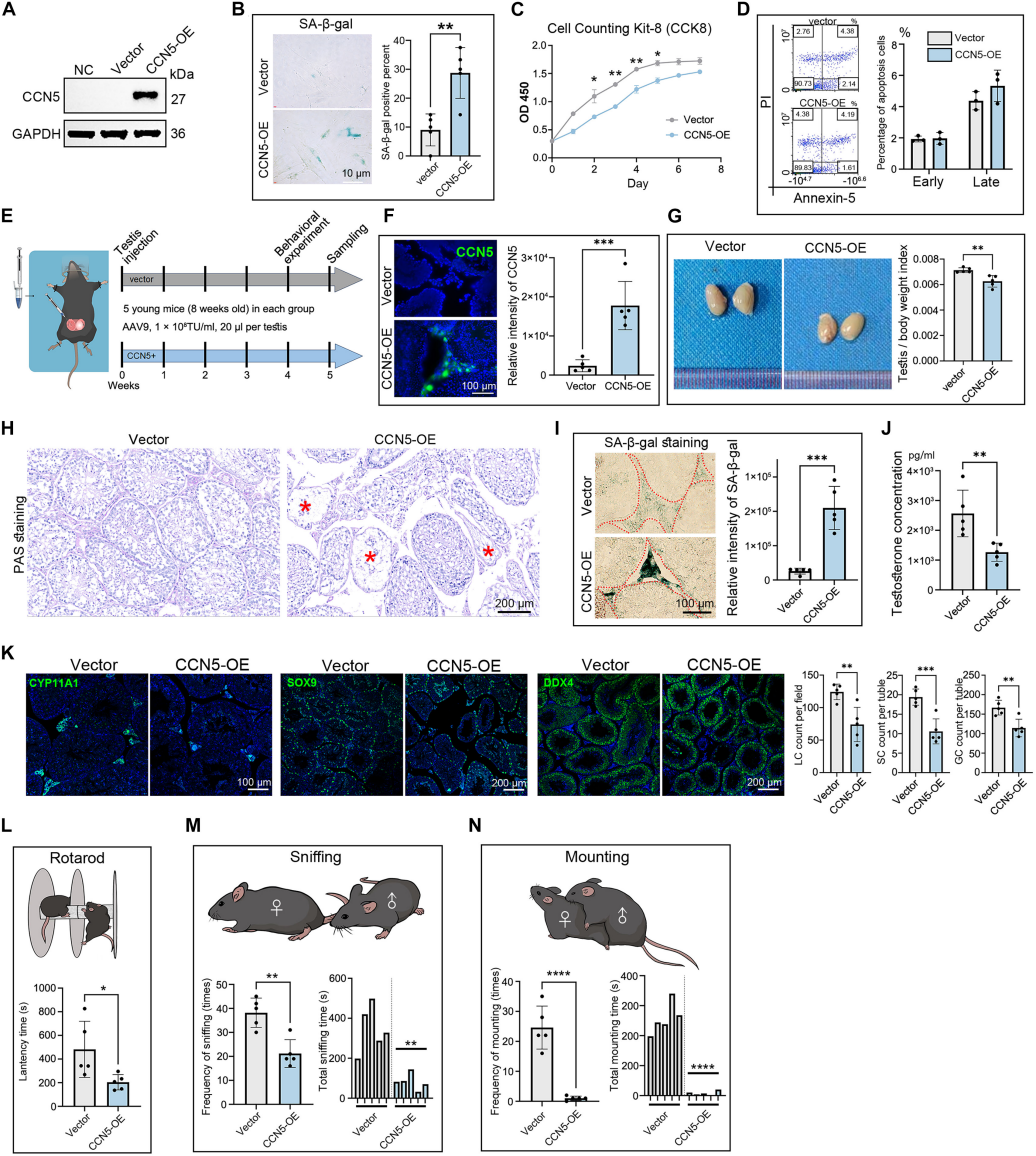
CCN5 overexpression leads to LC senescence and a decline in testicular function. (A) Western blot shows the CCN5 protein level of LCs before and after transfected with CCN5 overexpression or control plasmids. (B) SA-β-gal staining of LCs transfected with CCN5 overexpression or control plasmids. The scale bar represents 10 μm. *n* = 4 technical repetitions. (C) CCK-8 assay shows the proliferation of LCs transfected with CCN5 overexpression or control plasmids. *n* = 5 technical repetitions. (D) Flow cytometry demonstrated the influence of LCs transfected with CCN5 overexpression or control plasmids on the early (Q4) and late apoptosis (Q2) of LCs. The existence of Annexin V (*x*-axis) and the nuclear staining of PI (*y*-axis) by flow cytometry were shown. *n* = 3 technical repetitions. (E) Schematic illustration of the experimental workflow in vivo. (F) Immunofluorescence staining of CCN5 in testis after injection with CCN5-AAV9 or vector. The scale bar represents 100 μm. The right panel shows the fluorescence intensity statistics. *n* = 5 biological independent samples. (G) Testis after injection with CCN5-AAV9 showed a lower testis/body weight index. *n* = 5 biological independent samples. (H) ELISA results showing the testosterone concentration in the supernatant of cultured LC. *n* = 5 biological independent samples. (I) PAS staining of testis after injection with CCN5-AAV9 or vector. Asterisks indicate severely atrophied and degenerated seminiferous tubules. The scale bar represents 200 μm. (J) SA-β-gal staining of testis after injection with CCN5-AAV9 or vector. The scale bar represents 100 μm. *n* = 5 biological independent samples. (K) Immunofluorescence staining of CYP11A1 (LCs marker), SOX9 (SC marker), or DDX4 (germ cell marker) in testis after injection with CCN5-AAV9 or vector. The scale bar represents 200 μm. *n* = 5 biological independent samples. (L) The rotarod is used to assess endurance in mice. *n* = 5 biological independent samples. (M and N) Sexual behaviors including sniffing (M) and mating (N) in mice injection with CCN5-AAV9 or vector. *n* = 5 biological independent samples.

To functionally validate the relationship between CCN5 and testicular aging, we transfected CCN5-expressing plasmids into young cultured LCs (Fig. 3A). The up-regulation of CCN5 significantly increased SA-β-gal staining positivity and reduced cell proliferation capacity (Fig. 3B and C). However, CCN5 overexpression did not increase LCs apoptosis **(**Fig. 3D**)**.

To further investigate the role of CCN5 in vivo, we used AAV9 to transfect CCN5 overexpression plasmids into the testes of young mice, and successfully elevating CCN5 protein levels in the testicular interstitial region (Fig. 3E and F). Compared to controls, mice overexpressing CCN5 exhibited a slight decrease in testis organ index, caused more seminiferous tubules atrophy, and a significant increase in SA-β-gal activity in interstitial regions (Fig. 3G to I). Functionally, CCN5 overexpression led to a significant reduction in testosterone production of testicular tissue (Fig. 3J). Immunofluorescence staining further indicated that CCN5 overexpression significantly decreased the numbers of LC, Sertoli cells, and germ cells in the testis, highlighting CCN5’s extensive impact on the entire testicular microenvironment (Fig. 3K).

Testicular aging affects reproductive endocrine homeostasis, potentially leading to reduced physical endurance, decreased libido, and impaired fertility in males [1,29]. Behavioral assays revealed that mice overexpressing CCN5 spent less time on the rotarod (indicating reduced physical stamina) and exhibited significantly less sniffing and climbing behavior toward estrous female mice housed with them (Fig. 3L to N). These findings demonstrate that CCN5 overexpression induces aging and functional disruption across the testicular microenvironment, impacting individual physical strength and sexual desire.

### CCN5 induces LCs’ aging phenotypes through distinct pathways

**Fig. 4.**
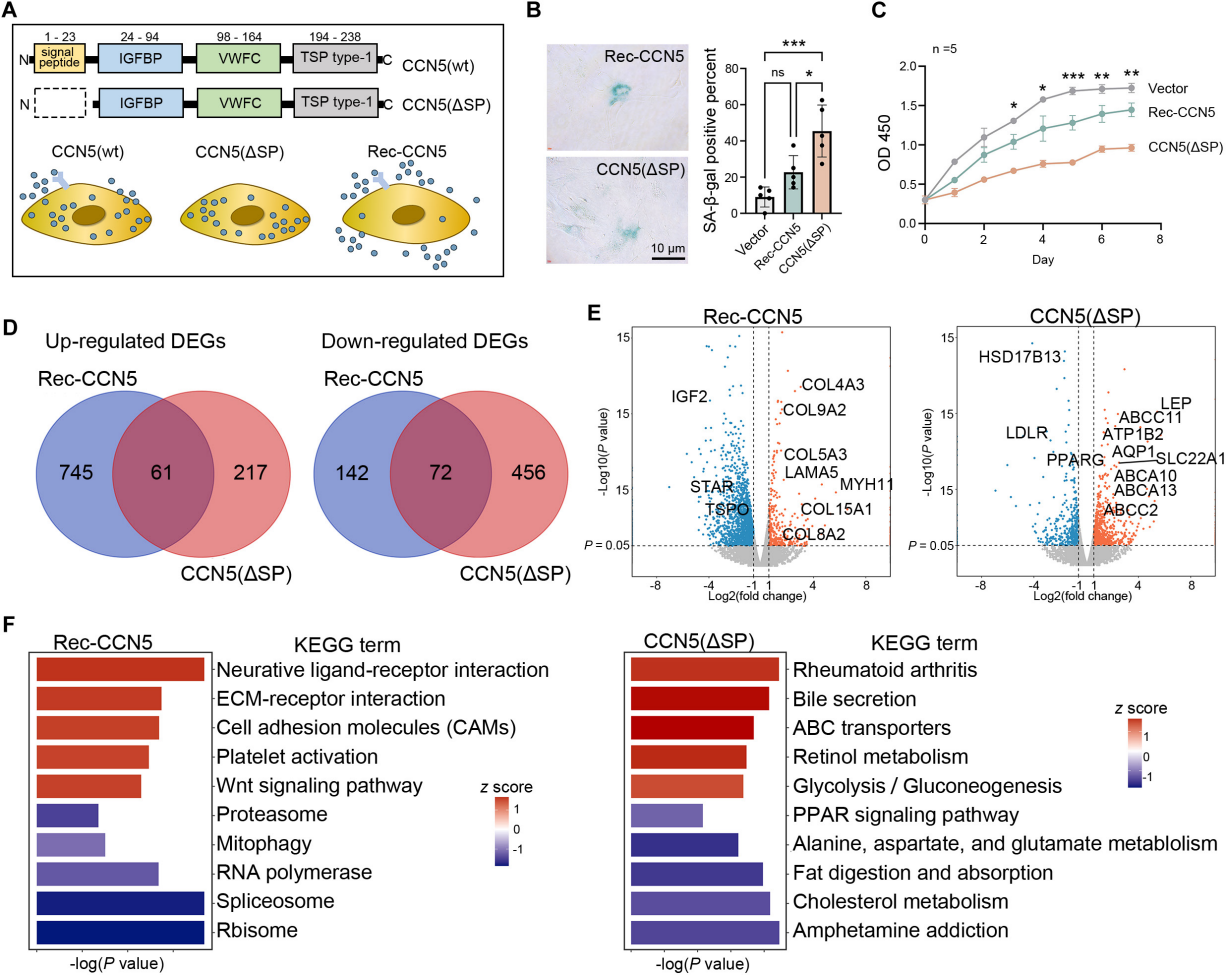
CCN5 induces different changes in LC phenotypes through intracellular and extracellular pathway. (A) Schematic diagram of the experimental grouping and treatment in this section. (B) SA-β-gal staining of LCs with CCN5(ΔSP) overexpression or recombinant CCN5 protein incubation. The scale bar represents 10 μm. *n* = 5 technical repeats. (C) CCK-8 assay shows the proliferation of LCs with CCN5(ΔSP) overexpression or recombinant CCN5 protein incubation. (D) The Venn diagram shows the overlap of up-regulated (left panel) or down-regulated (right panel) genes in the CCN5(ΔSP) overexpression and recombinant CCN5 protein treatment groups. (E) Volcano plot shows the DEGs in the CCN5(ΔSP) overexpression (left panel) and recombinant CCN5 protein treatment groups (right panel). (F) Bar plot showing the GSEA (KEGG pathways) terms according to the DEGs of LCs after CCN5(ΔSP) overexpression or recombinant CCN5 protein incubation.

As a secreted protein, CCN5 has been reported to influence ECM remodeling and cellular metabolism, corresponding to fibrosis and lipid droplet reduction, the 2 aging phenotypes of LCs [17,18,20,30]. It has been reported that CCN5 may regulate downstream signaling pathways through membrane receptors and transcription factors [20]. Therefore, we used plasmids for wild-type CCN5 and a signal peptide-deficient CCN5(ΔSP), where the latter lacks the N-terminal amino acids 1 to 23 and thus cannot be secreted, enabling us to simulate the intracellular effects of CCN5. Additionally, we added recombinant CCN5 protein directly to the cell culture medium to mimic its extracellular effects (Fig. 4A).

Interestingly, both CCN5(ΔSP) and recombinant CCN5 led to an increase in SA-β-gal staining positivity and a decrease in cell viability in LC, though recombinant CCN5 protein incubation only resulted in a slight reduction in LC viability (Fig. 4B and C). To further explore the distinct intracellular and extracellular effects of CCN5 on LC, we performed transcriptome sequencing and analyzed the DEGs in both Rec-CCN5 and CCN5(ΔSP) groups, finding that the overlap between them were small (less than 50%), suggesting that the 2 modes of CCN5 action regulate different downstream genes (Fig. 4D and Table S3). Focusing on genes related to fibrosis and lipid metabolism, we observed that fibrosis-related genes were predominantly enriched in the up-regulated genes of the Rec-CCN5 group, while lipid metabolism-related genes were enriched in those up-regulated by CCN5(ΔSP) (Fig. 4E). Gene set enrichment analysis (GSEA) revealed that Rec-CCN5 treatment led to the activation of the “ECM–receptor interaction” and “Wnt signaling pathway”; however, these changes were absent in the CCN5(ΔSP) group. Instead, we noted the change related to lipid metabolism in CCN5(ΔSP) group such as activation of the “ABC transporters”, and inhibition of the“Cholesterol metabolism” and “Fat digestion and absorption” terms (Fig. 4F). These results collectively suggest that CCN5 exerts its effects on LCs through distinct molecular mechanisms depending on its intracellular versus extracellular localization.

### Extracellular CCN5 induces LC fibrosis via the WNT-SMAD2/3 signaling axis

**Fig. 5.**
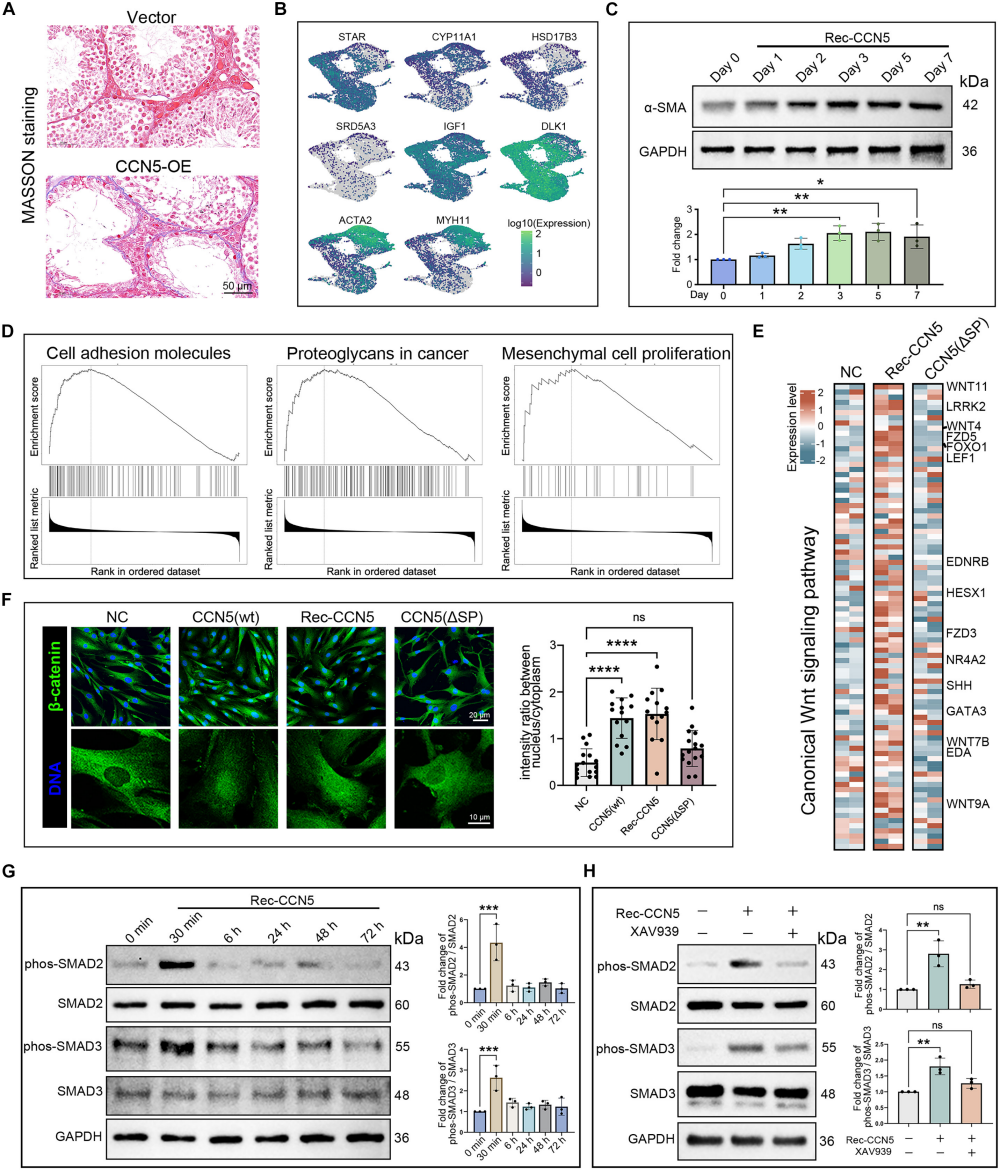
Extracellular incubation with recombinant CCN5 protein induces a fibrotic phenotype in LCs. (A) UMAP plots of LC cluster. Cells are colored according to the expression level of specific gene. (B) Masson's staining of mice testis tissue transfected with CCN5 overexpression or control plasmids. The scale bar represents 50 μm. (C) Western blot shows the dynamic change of α-SMA (ACTA2) protein in LCs after recombinant CCN5 protein incubation. *n* = technical repeats. (D) Heatmap shows the expression pattern of WNT signal related genes in LCs with CCN5(ΔSP) overexpression or recombinant CCN5 protein incubation. Genes with significant change are listed. Statistical analysis made by 2-tailed, ANOVA test; the confidence interval is 95%. (E) Immunofluorescence of β-catenin in cultured LCs with CCN5(ΔSP) overexpression, CCN5(wt) overexpression, or recombinant CCN5 protein incubation. The bottom panel is a local magnification of the top panel. The scale bar represents 10 μm (bottom panel) and 20 μm (top panel). *n* = 15 cells. (F) GSEA with the DEGs of LCs after recombinant CCN5 protein incubation. (G) Western blot shows the dynamic change of SMAD signal proteins in LCs after recombinant CCN5 protein incubation. *n* = technical repeats. (H) Western blot shows the expression level of SMAD signal proteins in LCs with or without recombinant CCN5 protein incubation and XAV939.

In this section, we first confirmed that CCN5 overexpression leads to a fibrosis phenotype in LCs similar to what is observed in aging testes (Fig. 5A). Our analysis of the LCs cluster aging trajectory showed a progressive decline in the expression of steroid hormone-metabolizing enzymes, accompanied by an increase in *ACTA2* and *MYH11* expression (Fig. 5B). In vitro, incubation with Rec-CCN5 led to a time-dependent increase in ACTA2 protein levels in LCs (Fig. 5C). GSEA further revealed significant activation of several fibrosis-related terms, such as “Cell adhesion molecules”, “Proteoglycans in cancer”, and “Mesenchymal cell proliferation” in the Rec-CCN5 incubation group (Fig. 5D). As a member of the CCN protein family, CCN5 is known to interact with WNT signaling and affects tissue fibrosis [17,21,31]. Transcriptome analysis revealed that multiple genes within the canonical WNT signaling pathway were up-regulated by Rec-CCN5 but not by CCN5(ΔSP), suggesting that only extracellular CCN5 activates WNT signaling (Fig. 5E). Immunocytochemical staining demonstrated a notable increase in nuclear β-catenin in both CCN5(wt) overexpression and Rec-CCN5 incubation groups, but not in the CCN5(ΔSP) group (Fig. 5F). We similarly observed an age-associated increase in β-catenin levels within the human testicular interstitial region (Fig. S1C). The Wnt/β-catenin signal is synergistic with the cytoskeleton change and TGF-β/SMADs pathway to promote tissue fibrosis [32,33]. We observed a transient increase (within 30 min) of SMAD2 and SMAD3 phosphorylation in LCs incubated with Rec-CCN5, and this effect was inhibited by the WNT pathway inhibitor XAV939 (Fig. 5G and H). These findings indicate that CCN5 induces fibrosis in an extracellular WNT-dependent manner by activating SMAD2/3.

### Intracellular CCN5 regulates the cholesterol transport of LCs

**Fig. 6.**
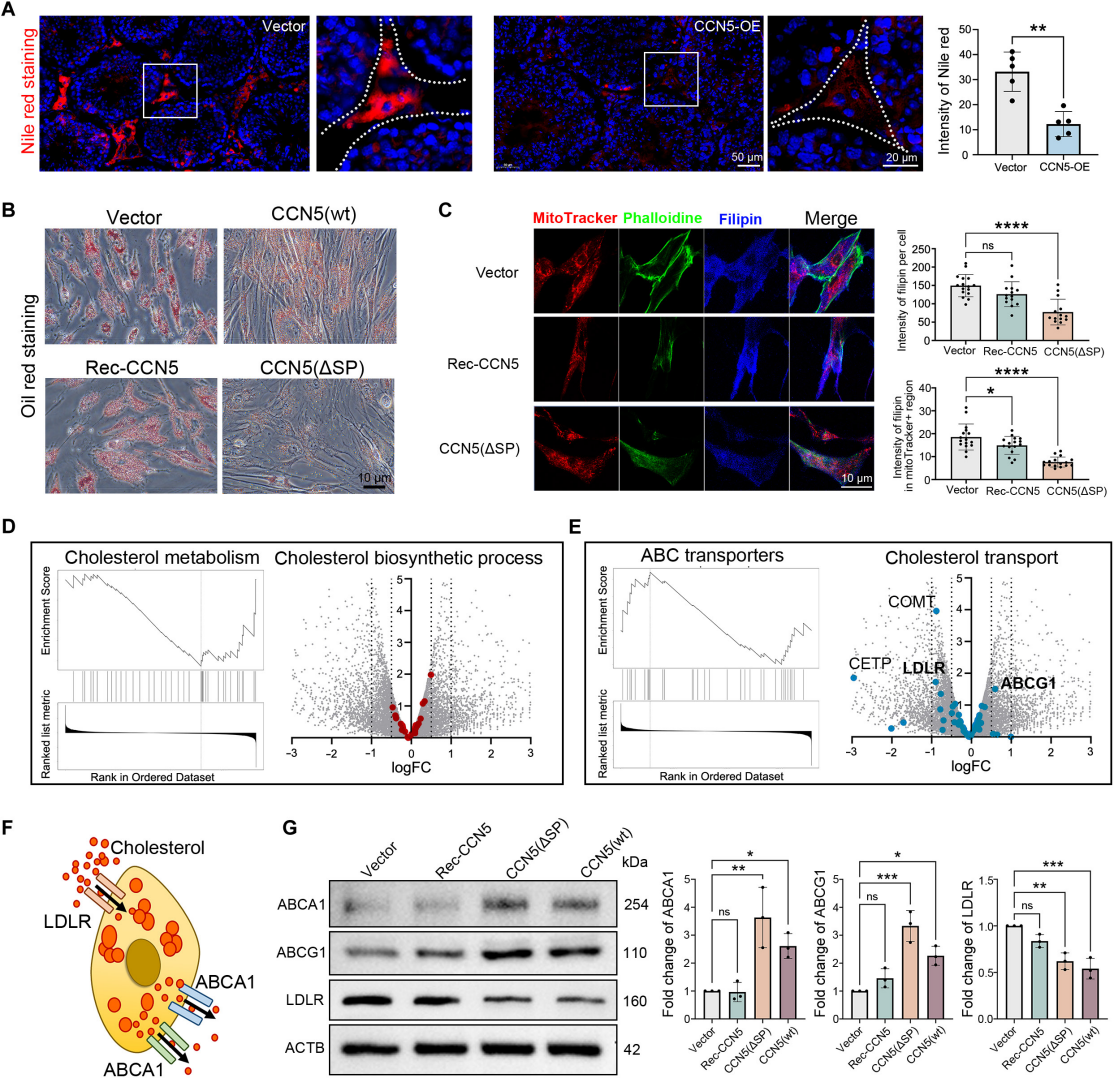
Overexpression of CCN5(ΔSP) disrupts cholesterol metabolism in LCs. (A) Nile red staining of mice testis tissue transfected with CCN5 overexpression or control plasmids. The scale bar represents 20 μm or 50 μm. *n* = 5 biological independent samples. (B) Immunofluorescence of β-catenin in cultured LCs with CCN5(ΔSP) overexpression, CCN5(wt) overexpression, or recombinant CCN5 protein incubation. The scale bar represents 10 μm. (C) Immunofluorescence co-staining of Mitotracker (red), phalloidin (green), and filipin (blue) in cultured LCs with CCN5(ΔSP) overexpression or recombinant CCN5 protein incubation. The scale bar represents 10 μm. *n* = 15 cells. (D) GSEA of “cholesterol metabolism” and volcano plot of “cholesterol biosynthetic process” term depend on the DEGs of LCs with CCN5(ΔSP) overexpression. (E) GSEA of “ABC transporters” and volcano plot of “cholesterol transport” term depend on the DEGs of LCs with CCN5(ΔSP) overexpression. (F) Schematic representation of cellular cholesterol uptake and efflux. (G) Western blot shows the expression of cholesterol transporters in LCs with CCN5(ΔSP) overexpression, CCN5(wt) overexpression or recombinant CCN5 protein incubation. *n* = 3 technical repeats.

Lipid droplet reduction is another key feature of LCs’ aging (Fig. 1H). Using Nile Red staining, we observed that CCN5(wt) overexpression leads to a decrease in lipid droplet in the testicular interstitial area of young mice (Fig. 6A). In vitro, overexpression of CCN5(ΔSP) significantly reduced lipid droplets in LC, whereas Rec-CCN5 incubation had minimal effect (Fig. 6B). Considering that the initial step of testosterone synthesis requires cholesterol transport to the mitochondrial inner membrane, we used MitoTracker and filipin to label mitochondria and cholesterol, respectively. The results indicated that both total cellular cholesterol content and mitochondrial cholesterol were significantly reduced in the CCN5(ΔSP) overexpression group (Fig. 6C). Transcriptome sequencing also revealed that the cholesterol metabolism pathway was generally down-regulated in the CCN5(ΔSP) overexpression group (Fig. 6D). These findings support the hypothesis that CCN5 depletes testosterone synthesis precursors through intracellular rather than extracellular mechanisms.

To further investigate the cause of cholesterol reduction in LC, we examined both de novo cholesterol synthesis and its extracellular uptake. We found no significant changes in genes related to de novo synthesis, but cholesterol transport-related genes were altered (Fig. 6D and E). In LCs, low-density lipoprotein receptor, associated with cholesterol uptake, was significantly down-regulated in both the CCN5(ΔSP) and CCN5(wt) overexpression groups (Fig. 6F and G). Conversely, cholesterol efflux transporters, such as ATP binding cassette subfamily A member 1 (ABCA1) and ATP binding cassette subfamily G member 1 (ABCG1), were significantly up-regulated in the CCN5(ΔSP) and CCN5(wt) groups, with no effect from Rec-CCN5 incubation (Fig. 6F and G). These results indicate that intracellular CCN5 reduced cholesterol levels in LCs through regulating cholesterol transport protein expression.

### CCN5 mediates LCs’ aging by binding and down-regulating RNF213

**Fig. 7.**
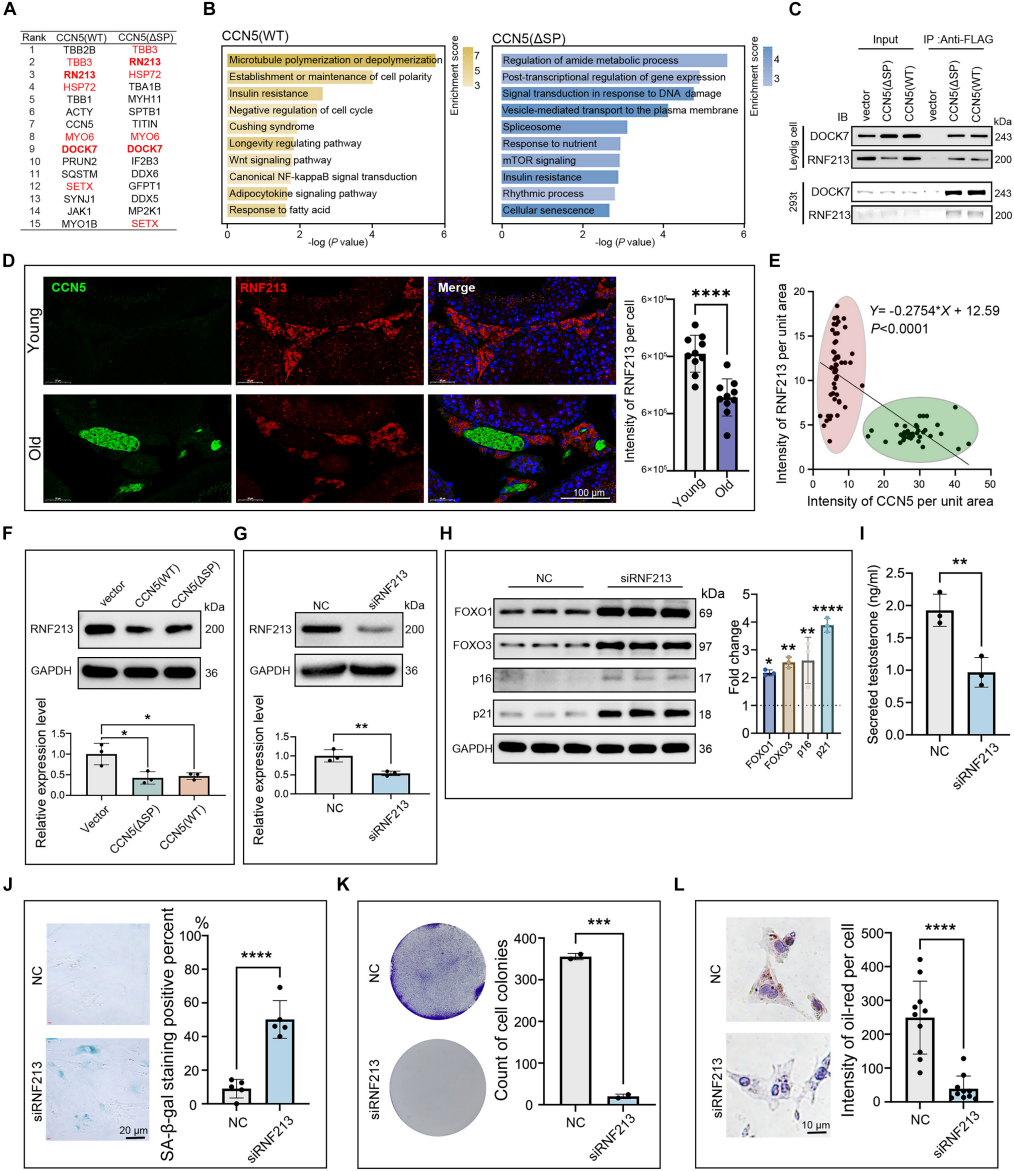
CCN5 promotes LCs senescence by down-regulating RNF213. (A) This table shows the top 10 proteins that combined with CCN5 identified by LC-MS/MS. (B) Bar plot shows the enrichment analysis of proteins that combined with CCN5. (C) The LCs (top panel) and 293t (bottom panel) cells lysates were immunoprecipitated by anti-FLAG antibody (fused with CCN5) or IgG with protein A/G magnetic beads. The precipitated proteins were analyzed by Western blot using anti-DOCK7 and anti-RNF213 antibody, respectively. (D) Immunofluorescence co-staining of RNF213 (red) and CCN5 (green) in young and old mice testis. The scale bar represents 100 μm. *n* = 10 different regions. (E) Scatter plot showing the correlation between CCN5 and RNF213 expression levels in the testicular interstitial region. (F) Western blot shows the level of RNF213 in LCs after recombinant CCN5 protein with CCN5(ΔSP) or CCN5(wt) overexpression. *n* = 3 technical repeats. (G) Western blot analysis to validate the knockdown efficiency of RNF213 in LCs. *n* = 3 technical repeats. (H) Western blot shows the fold change of FOXO1, FOXO3, p16, and p21 in LCs after knockdown of RNF213. *n* = 3 technical repeats. (I) ELISA was used to measure the change of testosterone concentration in the culture supernatant of LCs after knockdown of RNF213. *n* = 3 technical repeats. (J) SA-β-gal staining of LCs with RNF213 knockdown. The scale bar represents 20 μm. *n* = 5 technical repeats. (K) Crystal violet staining marks colony formation in LCs with RNF213 knockdown. *n* = 2 technical repeats. (L) Oil red staining of LCs with RNF213 knockdown. The scale bar represents 10 μm. *n* = 10 cells.

Next, we explored the specific mechanisms through which intracellular CCN5 induces LCs’ aging. Using protein immunoprecipitation coupled with liquid chromatography–tandem mass spectrometry (LC-MS/MS) analysis, we identified the top proteins binding to both CCN5(ΔSP) and CCN5(wt), and also found a substantial overlap between them (Fig. 7A and Table S4). Proteins binding to CCN5(wt) were enriched in lipid metabolism related terms such as “insulin resistance”, “Cushing syndrome”, “Longevity regulating pathway”, and “response to fatty acid”, while those binding to CCN5(ΔSP) were enriched in terms related to "response to nutrient", “insulin resistance”, and “cellular senescence” (Fig. 7B). Among these proteins, DOCK7 and RNF213 were identified as high-confidence CCN5-binding proteins, with this binding effect also observed in other cell types, such as 293T cells (Fig. 7C).

In vivo, DOCK7 and RNF213 proteins are both localized in the testicular interstitial area, RNF213 expression decreases with aging in human testis tissues (Fig. S2A), while DOCK7 expression is higher in aged mice testis (Fig. 7D and Fig. S2B). In addition, RNF213 and CCN5 expression levels exhibit an inverse relationship within the same region of testicular tissue (Fig. 7E). This hypothesis was confirmed by WB test, which showed that overexpression of both CCN5(wt) and CCN5(ΔSP) reduced RNF213 levels (Fig. 7F).

RNF213, a unique E3 ubiquitin ligase, is associated with inflammatory responses and lipid droplet metabolism, all of which align with the aging phenotypes observed in LCs [34,35]. To validate this, we knocked down RNF213 in LCs using siRNA (Fig. 7G). Immunoblotting revealed that RNF213 knockdown activated forkhead box O (FOXO) 1/3 and up-regulated aging markers p16 and p21 (Fig. 7H). In addition, RNF213 knockdown also reduced the testosterone synthesis, colony formation, and intracellular lipid droplet in LCs while significantly increasing SA-β-gal activity (Fig. 7I to L).

Additionally, DOCK7 has been reported to be involved in cell cycle regulation and lipid metabolism [36,37]. We found that knockdown of DOCK7 caused a slight increase in lipid droplet content (Fig. S2C). However, no significant changes were noted in colony formation or SA-β-gal activity, suggesting that DOCK7 is not a primary pathway for CCN5-induced senescence (Fig. S2D and E).

### Down-regulation of CCN5 expression restored testicular function in aged mice

**Fig. 8.**
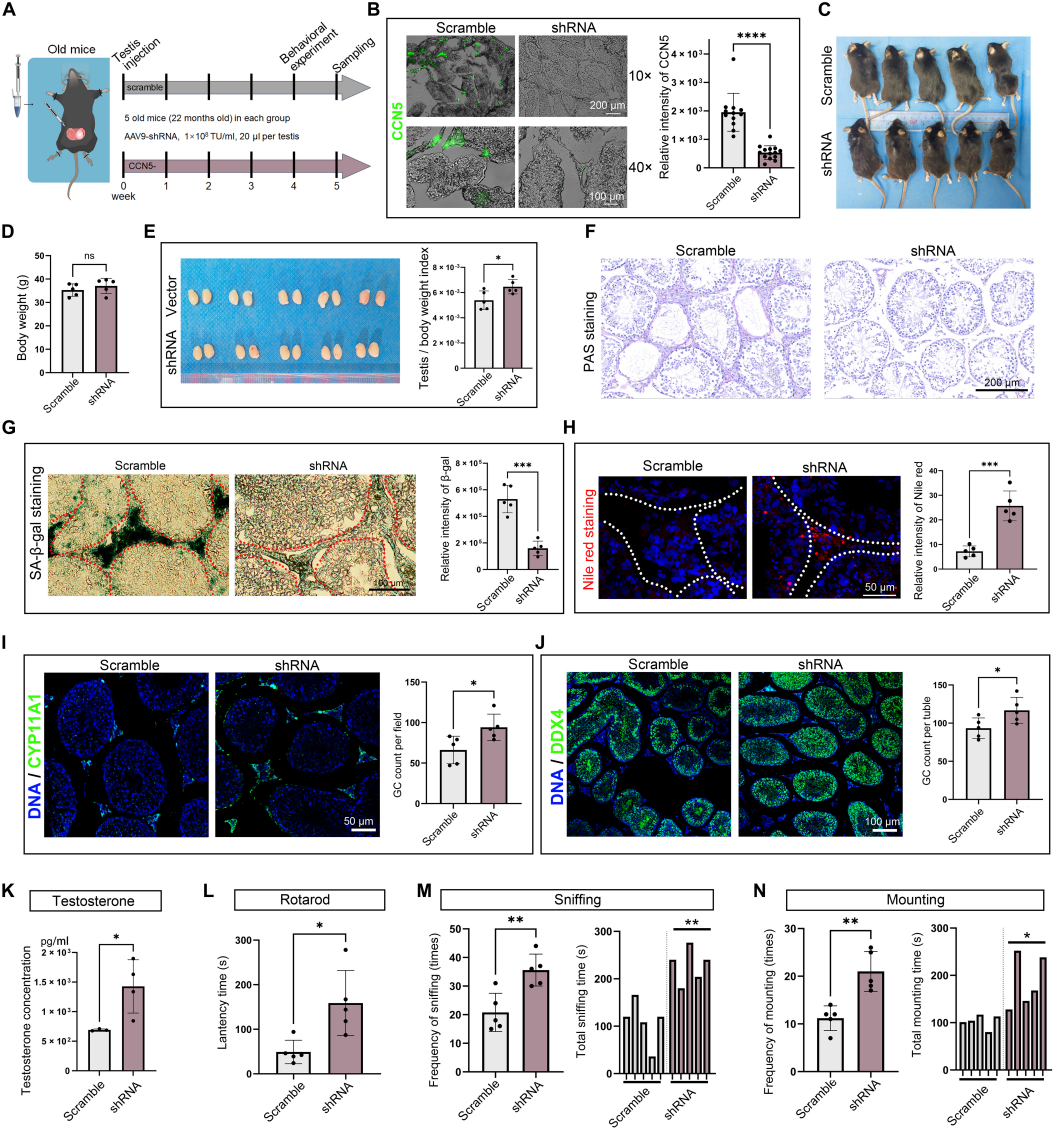
Knockdown of CCN5 in vivo alleviates age-related testicular degeneration in aged mice. (A) Schematic illustration of the experimental workflow in vivo. (B) Immunofluorescence staining of CCN5 in testis after injection with shRNA of CCN5 or scramble. The scale bar represents 100 μm (bottom panel) or 200 μm (top panel). *n* = 10 regions of 5 biological independent samples. (C) Photograph of aged mice after testicular injection of shRNA-AAV. (D) The body weight of aged mice after testicular injection of shRNA-AAV. *n* = 5 biological independent samples. (E) Photograph of testis from aged mice after testicular injection of shRNA-AAV. Testis/body weight index data from 5 biological independent samples. (F) PAS staining of testis from aged mice after testicular injection of shRNA-AAV. The scale bar represents 200 μm. (G) SA-β-gal staining of testis from aged mice after testicular injection of shRNA-AAV. The scale bar represents 100 μm. *n* = 5 biological independent samples. (H) Nile red staining of mice testis tissue after injection with shRNA-AAV of CCN5 or scramble. The scale bar represents 50 μm. *n* = 5 biological independent samples. (I and J) Immunofluorescence staining of (I) CYP11A1 (LCs marker) or (J) DDX4 (germ cell marker) in testis after injection with shRNA-AAV of CCN5 or scramble. The scale bar represents (I) 50 μm or (J) 100 μm. *n* = 5 biological independent samples. (K) ELISA results showing the testosterone concentration in the testicular tissue homogenate from aged mice after testicular injection of shRNA-AAV. *n* = 5 biological independent samples. (L) The rotarod is used to assess endurance in mice. *n* = 5 biological independent samples. (M and N) Sexual behaviors including sniffing (M) and mating (N) in mice after injection with shRNA-AAV or scramble. *n* = 5 biological independent samples.

Next, we explored whether targeting CCN5 expression could improve testicular aging. To achieve this, we locally injected AAV9-shRNA into the testis of aged mice to knock down CCN5 expression (Fig. 8A and B). Although shRNA treatment did not affect body weight, it significantly increased the testis/organ index in aged mice (Fig. 8C to E). Periodic acid schiff (PAS) staining indicated improved seminiferous tubule atrophy in the shRNA treatment group (Fig. 8F). In addition, the SA-β-gal activity significantly decreased in the testicular interstitial regions, while Nile red staining signals, indicating lipid droplet presence, increased (Fig. 8G and H). Additionally, the numbers of LCs and germ cells were elevated by CCN5 knockdown (Fig. 8I and J). Most notably, CCN5 knockdown effectively restored testosterone synthesis in aged testes (Fig. 8K). Subsequent behavioral studies showed an increase in time kept on the rotarod, as well as more frequent and prolonged sniffing and mounting behaviors toward females (Fig. 8L to N). However, no pregnancies were observed in either the treatment or control groups during co-housing with females, suggesting that sperm quality in aged mice may be affected by other factors. In summary, our research identifies CCN5 as a biomarker of testicular aging and demonstrates the feasibility of targeting CCN5 to improve testicular function in aged mice.

## Discussion

The senescence of LCs is the most direct cause of testicular dysfunction and reproductive endocrine disorders in elderly men. Due to the complications associated with exogenous testosterone supplementation, such as testicular atrophy, sexual dysfunction, and an increased risk of cardiovascular events, giving endogenous testosterone production represents a more promising therapeutic approach for LOH [13]. To identify biomarkers of LCs’ aging, we integrated multiple human testicular scRNA-seq datasets to construct a developmental and aging trajectory of the testis in males aged 0 to 80 [10,12,13,23]. We intersected LC-specific expressed genes, senescence-up-regulated genes, and genes highly expressed in disease with premature aging phenotypes, such as iNOA [12]. Among these, CCN5 was identified as a critical candidate closely associated with ECM remodeling and metabolic regulation, both of which align with key features of LCs’ aging: fibrosis and lipid droplet loss [17,20,30]. Serum CCN5 levels also correlated strongly with age [38]. In addition, CCN5 is highly enriched in the testis (https://www.proteinatlas.org/ENSG00000064205-CCN5/tissue), suggesting that serum CCN5 may serve as a specific biomarker for testicular aging [39].

We demonstrated in both in vivo and in vitro experiments that CCN5 drives multiple senescence phenotypes in LCs; it appeared to be beyond the explanation of a single signaling pathway. As a member of the Wnt-1-inducible signaling pathway protein (WISP) family, CCN5 expression is regulated by Wnt signaling [40]. However, the relationship appears to be bidirectional, as CCN5 overexpression activated β-catenin, while treatment with the canonical Wnt inhibitor DKK1 antagonized CCN5’s effects [19,41]. Wnt signaling has been reported to negatively regulate testosterone synthesis, and its inhibition is necessary for LC maturation [42]. Aberrant Wnt activation impairs testicular development and is enriched in conditions such as iNOA [43]. Wnt signaling is also a potent profibrotic factor involved in age-related fibrosis of the kidney, liver, and skin [44]. Our study showed that extracellular CCN5 activates SMAD2/3, 2 fibrosis-related transcription factors, via a Wnt-dependent mechanism. ECM remodeling is another hallmark of tissue fibrosis, and CCN5 has been reported to bind collagen, influencing collagen fiber alignment and distribution [18]. Interestingly, in addition to the 2 proteins RNF213 and DOCK7 reported here, we also found that CCN5 bound to a variety of cytoskeletons, such as MYH9, ACTG, and TPM3, which may similarly induce the transition of LCs to a myofibroblast phenotype through biomechanical changes [45].

In contrast, extracellular CCN5 had no significant effect on lipid metabolism in LCs, which was mediated instead through intracellular CCN5. In adipocytes, CCN5 interacts with and inhibits ZFP423 nuclear translocation, activating peroxisome proliferator-activated receptor gamma (PPARγ) and committing cells to the adipocyte lineage [20]. Similarly, CCN5(ΔSP) overexpression in LCs reduced PPARγ transcription, potentially disrupting cholesterol transporter balance [20]. In LCs, we did not detect ZFP423 in the CCN5 protein complex, but another lipid-regulating protein, RNF213, drew our attention. RNF213 competes with adipose triglyceride lipase on lipid droplets, and its reduction destabilizes lipid droplets, potentially leading to cholesterol efflux [35]. With the advancement of technology, it is expected to identify the specific effects of CCN5 on lipid homeostasis of LCs at the single-cell level in the future, which may help to develop non-invasive diagnostic methods for testicular aging [46]. Interestingly, our study revealed that knockdown of RNF213 unexpectedly increased SA-β-gal positivity and induced cell cycle arrest in LCs, a finding contrary to its role observed in tumors [47]. This discrepancy might be attributed to the distinct pro- or anti-aging effects of FOXO1/3 in different tissues. For instance, in aged hepatocytes of mice, elevated FOXO1 expression has been shown to impair glucose tolerance, and suppression of FOXO1 attenuates inflamm-aging and improves liver function [48]. Conversely, in synovial cells, FOXO1 protects against cellular aging by reducing the secretion of inflammatory cytokines and chemokines [49]. These findings suggest that CCN5 may serve as a more specific therapeutic target for testicular aging compared to FOXOs.

Previous literature indicates that CCN5 may have a dual effect of pro-fibrosis and anti-fibrosis. In the liver, CCN5 expression elevated in damaged hepatocytes, then interacts with specific integrin subtypes to promote liver fibrotic progression [50,51]. These results are consistent with our observations during the aging process of the testicles. However, CCN5 also shows anti-fibrotic effects in cardiac and pulmonary tissues [52]. In addition, CCN5 has been reported to promote islet cell proliferation and survival in vitro, and it seems to contradict the pro-senescence effect of CCN5 on LCs, as senescent cells typically undergo cell cycle arrest [53,54]. We speculate that the effect of CCN5 in controlling tissue fibrosis and cell cycle may be related to the cell type and the state of the tissue microenvironment, and even Wnt pathway heterogeneity may also play an important role. This mechanistic divergence underscores the critical importance of testicular-targeted drug delivery over systemic administration. Local intra-testicular administration confined by the blood–testis barrier could prevent unintended interference with fibrotic processes in other organs, whereas systemic delivery risks disrupting extra-testicular fibrotic homeostasis through CCN5’s pleiotropic effects. Our approach therefore prioritizes spatial precision in therapeutic intervention to mitigate potential off-target consequences.

This study has several limitations. First, we did not collect sufficient human blood samples to determine whether CCN5 concentration correlate with testosterone or other testicular aging biomarkers, which limits the diagnostic value of CCN5. Second, the specific cell membrane receptor involved in CCN5’s extracellular pathway remains unclear. Previous reports suggest LRP5/6 as a potential candidate; however, we did not detect them within the CCN5 protein complex [55]. Lastly, the mechanism by which intracellular CCN5 leads to RNF213 down-regulation is still unknown and requires further investigation through molecular biology experiments.

In summary, this study identifies CCN5 as a novel biomarker for testicular aging and demonstrates that CCN5 promotes LCs fibrosis and lipid droplet loss through distinct mechanisms involving the β-catenin/SMAD axis and RNF213 down-regulation, respectively. These findings highlight CCN5 as a promising therapeutic target for mitigating testicular aging and associated reproductive endocrine disorders.

## Conclusion

Based on the scRNA-seq aging trajectory of LCs, we identified fibrosis and intracellular lipid droplet loss as the 2 main characteristics of LCs’ aging. The expression level of CCN5 was significantly elevated in aging LCs. Overexpression of CCN5 accelerated the onset of aging phenotypes in young LCs and led to testicular functional decline. Extracellular CCN5 activated β-catenin signaling and then promoted SMAD2/3 phosphorylation, leading to the fibrotic phenotype in LCs. Intracellularly, CCN5 bound to and down-regulated RNF213 protein level, resulting in lipid droplet degradation. Intracellular CCN5 disrupts the balance between cholesterol influx and efflux by regulating the expression of cholesterol transport proteins. Additionally, CCN5 binds to and down-regulated RNF213, leading to lipid droplet degradation. Up-regulated FOXO1/3 further induced the expression of p16 and p21, thereby promoting LC senescence-associated cell cycle arrest.

## Materials and Methods

### Human samples

The use of all human samples was approved by the ethics committee of the Fifth Affiliated Hospital of Sun Yat-sen University (license no. K278-1). The samples were obtained from testicular tissues that were resected or biopsied due to surgical necessity. The samples from young people were obtained from obstructive azoospermia biopsy surgeries of healthy individuals with normal hormone levels and normal spermatogenesis. Aged testicular tissue was sourced from patients undergoing orchidectomy for prostate cancer without receiving endocrine therapy (castration drugs) prior to surgery. The scRNA-seq data of human samples were obtained from publicly available datasets.

### Experimental animals

All animal experiments were approved by the Animal Ethics Committee of the Fifth Affiliated Hospital of Sun Yat-sen University (license no. 00359). The mice were maintained under standard housing conditions with a room temperature of 20 to 25 °C, humidity of 30%–70%, and a light cycle of 12 h. Wild-type mice on a C57BL/6 background were purchased from Guangdong Jinzhihe Biotechnology Co., Ltd. Naturally aged mice had free access to food and water, and their body weight was recorded weekly until they reached 80 weeks of age. The young male mice used in this study and the female mice used for mating behavior tests were both 8 weeks old and were purchased from Guangdong Jinzhihe Biotechnology Co., Ltd.

### scRNA-seq analysis

scRNA-seq data from testes of different ages were obtained from GSE149512, GSE215754, GSE182786, GSE143356, and GSE161617. The analysis and integration were performed using the Seurat package in R. Cells were further filtered based on the following threshold parameters: the total number of expressed genes, range 500 to 9,000; total unique molecular identifiers (UMI) count, range 0 to 35,000; and the proportion of mitochondrial gene expression, <40%. Normalization was performed according to the package manual (https://satijalab.org/seurat/v3.1/pbmc3k_tutorial.html). LC clusters were identified by the high expression of genes such as DLK1, CYP11A1, and IGF1. Cell pseudotime trajectories were constructed using the Monocle 3 package (version 1.3.1) according to its operation manual (https://cole-trapnell-lab.github.io/monocle3/docs/introduction/). Briefly, the UMI count matrices of the LC cluster were used as the expr_matrix, and meta.data was used as the sample sheet. Dimensionality reduction was performed using the reduce_dimension function with modified parameters: umap.n_neighbors = 25L and umap.min_dist = 0.2. The learn_graph function was used to construct the differentiation trajectories, with the learn_graph_control parameter set to 145. The starting point of the differentiation trajectory was set as the younger sample age group.

### Cell culture

Testis tissues were enzymatically digested with enzyme I (10 ml of DMEM containing 2 mg ml^−1^ type IV collagenase and 10 mg ml^−1^ DNase I) for 5 min, and primary LCs were isolated from the cell suspension according to the expression of NGFR by FACS. Then, a density of 2 × 10^5^ cells per milliliter of LCs were cultured on plastic dishes in 10% FBS DMEM expansion medium (contain 10 ng ml^−1^ LH and 2.5 μM cholesterol) at 37 °C in 5% CO_2_ for 24 h [13]. The murine LC line, TM3, was purchased from ZQXZ Bio (Catalog Number ZQ0100). The 293T cell line used as a tool in other experiments was obtained from ABM (Catalog Number T3251).

### Bulk RNA-seq of LC

A total of 1 × 10^5^ LCs from each experimental group were collected using TRIzol reagent (15596026; Thermo Fisher Scientific) for transcriptome sequencing. Library construction and high-throughput sequencing were performed in collaboration with Sinotech Genomics Co., Ltd (Shanghai, China). Total RNA extraction was carried out with the RNeasy Mini Kit (74904; Qiagen) following the manufacturer’s protocol. To ensure sequencing quality, poly(A)-containing mRNA was specifically purified using poly(T)-oligo-attached magnetic beads, and the isolated mRNA was subjected to fragmentation, reverse transcription, purification, and enrichment processes. Clustering was completed using the cBot system with the final library diluted to 10 pM for sequencing on the Illumina NovaSeq 6000 platform (Illumina, USA). The resulting paired-end FASTQ files were aligned to the mouse reference genome (mm39) using HISAT2 for accurate read mapping. Differential gene expression analysis was conducted with the R package edgeR, identifying significantly modulated mRNAs with thresholds of |log₂(FC)| > 1 and *q* < 0.05. These significantly DEGs were further visualized using volcano plots and subjected to Gene Ontology enrichment analysis to identify key biological processes. To validate the accuracy of the transcriptome sequencing results, the expression level of CCN5 was checked. Additionally, the purity of LCs was confirmed by analyzing the expression levels of well-established LC marker genes. These measures ensured the reliability and precision of the sequencing data in reflecting the transcriptional landscape of LC samples.

### Oil red staining

Fresh testicular tissue was washed twice with phosphate-buffered saline (PBS) to remove blood contamination and then fixed with 4% paraformaldehyde at 4 °C for 2 h prior to embedding in paraffin or freezing. Ten-micrometer sections were cut from optimal cutting temperature (OCT)-embedded samples using a cryostat, fixed in 4% paraformaldehyde, and rinsed in distilled water for two 3-min cycles. The sections were then submerged in 60% isopropanol for 20 to 30 s before being stained with oil red for 10 to 15 min. Nuclear counterstaining was performed using Mayer's hematoxylin for 1 to 2 min, after which the sections were washed under running tap water for 10 min or treated with a weak lithium carbonate solution for 3 min for bleaching. The sections were briefly rinsed in distilled water, blotted with filter paper to remove excess moisture, and finally mounted with glycerol gelatin or Arabic gum.

### Masson staining

The nuclei were stained with hematoxylin solution for 5 to 10 min, followed by rinsing under tap water for 10 to 15 min. Ponceau acid fuchsin solution was then used for staining for 5 to 10 min, after which the sections were dipped in 2% glacial acetic acid for 1 to 2 s. Differentiation was achieved using 1% phosphomolybdic acid aqueous solution for 3 to 5 min, followed by direct staining with aniline blue for 5 min. The sections were then rinsed in 0.2% glacial acetic acid for 1 to 2 s. For dehydration, the sections were processed through 70%, 90%, and 100% alcohol for 3 min, cleared with xylene for 5 min.

### H&E staining

Hematoxylin and eosin (H&E) staining was performed using the Biyun Tian H&E Staining Kit. The samples were stained with hematoxylin solution for 5 to 10 min, then rinsed under tap water for 10 to 15 min and washed once with distilled water. Eosin solution was applied for 1 to 2 min of staining. Following dehydration in 70%, 90%, and 100% ethanol for 3 min each, and clearing in xylene for 5 min, this process was repeated twice.

### Western blot

For Western blotting, LCs were seeded in 6-well plates and lysed with ice-cold RIPA buffer (Beyotime Biotechnology, P0013B) supplemented with protease and phosphatase inhibitors (Roche, 10390845). The lysates were incubated on ice for 15 min and then transferred to 1.5-ml Eppendorf (EP) tubes. The samples were centrifuged at 4 °C at 12,000 × *g* for 20 min. Proteins were separated by sodium dodecyl sulfate-polyacrylamide gel electrophoresis and transferred to nitrocellulose membranes for subsequent immunoblotting analysis with primary antibodies.

The following primary antibodies were used for immunoblotting: DYKDDDDK tag Monoclonal antibody (Proteintech, 66008-4-IG, 1:5,000), RNF213 Antibody (Novus, NBP1-88466, 1:1,000), Smad2 (D43B4) XP Rabbit mAb (CST, 5339T, 1:1,000), Phospho-SMAD2 (Ser465/Ser467) (E8F3R) Rabbit mAb (CST, 4511T, 1:1,000), Smad3 Rabbit pAb (Abclonal, A11471, 1:1,000), Phospho-Smad3-S423/S425 Rabbit mAb (Abclonal, AP0727, 1:1,000), alpha-SMA Antibody (Affinity Biosciences, AF1032, 1:1,000), ccn5-rabbit-polyclonal-antibody (Origene, TA383325; 1:1,000), and DOCK7 Polyclonal antibody (proteintech, 13000-1-AP, 1:1,000). Anti-Mouse IgG, horseradish peroxidase (HRP) (Cell Signaling Technology, 7074S; 1:3,000) and anti-Rabbit IgG, HRP (Cell Signaling Technology, 7076S; 1:3,000) were used as secondary antibodies.

### CCK-8 assay

The CCK-8 kit (Biosharp, catalog number cck8-BS350C-25*100T) was utilized to assess cell viability. Cells were seeded in a 96-well plate at a concentration of 2,000 cells per well in 2% FBS medium. Ten microliters of CCK-8 solution was added to each well, followed by incubation in the cell culture incubator for 1 h. Absorbance was then measured at 450 nm using a microplate reader.

### Flow cytometry for apoptosis

LC suspension was washed twice with cold PBS and resuspended in 1× binding buffer at a concentration of 1 × 10^6^ cells/ml. A 100-μl aliquot of the cell suspension, containing 1 × 10^5^ cells, was transferred to a 5-ml culture tube. The BD PMG Annexin V FITC Apoptosis Detect Kit I 100Tst (catalog number 556547) instructions were followed by adding 5 μl of FITC Annexin V and 5 μl of PI to the cells. After gentle vortexing, the cells were incubated at room temperature (25 °C) in the dark for 15 min. Subsequently, 400 μl of 1× binding buffer was added to each tube.

### Immunoprecipitation

Cells were collected and lysed in NP-40 lysis buffer containing a mixture of protease and phosphatase inhibitors. The lysates were centrifuged at 12,000 rpm at 4 °C for 15 min, and the supernatants were collected. Equal aliquots of the supernatant were incubated with a diluted FLAG primary antibody overnight at 4 °C, followed by the addition of protein A/G beads. The mixture was rotated at 4 °C for 4 h.

### LC-MS/MS

The protein solution collected from immunoprecipitation was subjected to protein electrophoresis. The gel containing the protein sample was stained using the Quick Silver Staining Kit (Beyotime, catalog number P0017S). Gel regions showing significant staining differences were excised and submitted for protein mass spectrometry analysis. The detection services and results were provided by Shenzhen Weinafei Biotechnology Co., Ltd.

### Testosterone measurement

For the measurement of testosterone within the testis, 20 mg of testicular tissue was accurately weighed and homogenized using a tissue grinder until no obvious tissue chunks remained in 500 μl of buffer (0.5% BSA, wt/vol, and 5 mM EDTA in PBS, pH 7.4). The tissue was further disrupted using a Q800R ultrasonic system (QSONICA). The homogenate was then centrifuged at 3,000 rpm at 4 °C for 10 min to separate the insoluble debris. The supernatant was collected for testosterone measurement. Testosterone levels were measured using a Total Testosterone ELISA Kit (abclonal, RK00724) according to the manufacturer’s instructions. Each sample was measured in triplicate wells, and the average value was taken after the measurements.

### Behavioral experiment

Sexual behavior. Female mice were prepared for the sexual behavior test through hormonal induction. Estradiol was administered 48 h before the test via intraperitoneal injection at a dose of 20 μg per mouse, dissolved in 0.1 ml of corn oil. Four hours prior to the test, each mouse received an intraperitoneal injection of 500 μg of progesterone, also dissolved in 0.1 ml of corn oil. The estrous stage of the female mice was confirmed using vaginal smears before initiating behavioral observations. To observe mating behavior, male mice were placed in the experimental cage before the introduction of the females. Under dim red light, estrous females were gently placed into the cage, and continuous video recording commenced, lasting 30 min. Sniffing behavior by the male mouse was defined as starting when the male approached the female’s tail and began sniffing around her genital or perianal area. Sniffing behavior was considered to end when the male turned away, stopped moving, or engaged in other non-sniffing behaviors. The number of sniffing incidents and the total duration of sniffing within the first 5 min after cohabitation were recorded and statistically analyzed. Mounting behavior was defined as the male climbing onto the female and performing rapid, shallow pelvic thrusts. Mounting episodes were considered to end when the male dismounted. The number of mounting incidents and the total duration of mounting within the 30-min observation period were recorded and analyzed.

Endurance test. Before the rotarod endurance test, mice underwent 7 sessions of adaptive training to ensure familiarity with the apparatus. For each formal experiment, mice were first placed on a rotating rod set at a constant speed of 5 rpm for 60 s to adapt. Afterward, the rod’s speed was increased to 20 rpm, and the duration each mouse could remain on the rotating rod was recorded over a 5-min period. Each test was performed 3 times, with a 15-min rest interval between trials. The maximum duration from the 3 trials was used for statistical analysis.

### Statistics and reproducibility

Unless otherwise specified, data are presented as mean ± SD. Statistical analyses for Figs. 2E, G, and H; 3B to D, F to H, and J to N; 6A; 7D, G, and I to L; and 8B, D, E, and G to N, and Fig. S2B, C, and E were performed using a 2-tailed unpaired Student’s *t* test with a 95% confidence interval. For Figs. 2F; 3B; 4C; 5C, E, G, and H; 6C and G; and 7F and H, statistical analyses were conducted using 2-tailed analysis of variance (ANOVA) followed by Tukey’s multiple comparisons test, also with a 95% confidence interval. Correlation statistical analysis for Fig. 7E was performed using a 2-tailed Spearman test with a 95% confidence interval. The significance levels in this study are denoted as follows: *P < 0.05, **P < 0.01, ***P < 0.001, ****P < 0.0001, and ns = no significant. For Fig. 2E, qPCR results were derived from 3 technical replicates. Figure 2F statistics were based on 5 different regions of a single human sample. The quantification of germ cells and Sertoli cells was expressed as the ratio of the number of cells to the number of seminiferous tubules, and the quantification of LCs was measured in units of the number under a 20× microscope field of view. Western blot results are based on 3 technical replicates. For other mouse experiments, statistical analyses were performed using at least 5 biological replicates.

## Data Availability

scRNA-seq data from testes of different ages were obtained from the GEO database with accession numbers GSE149512, GSE215754, GSE182786, GSE143356, and GSE161617. Bulk-RNA seq of human LCs with Rec-CCN5 treatment or CCN5(ΔSP) overexpression could be found in the GEO database with accession number GSE259443. For further information, resources, and reagents, please contact L.Z. (zhaoly37@mail.sysu.edu.cn).
