# Supplementary material for: CCN5 Drives Leydig Cell Aging and Testicular Dysfunction: Insights into Fibrosis, Lipid Dysregulation, and Therapeutic Potential
Source: Research (Wash D C). 2025 Aug 1;8:0762. doi: 10.34133/research.0762 (PMC12314281; doi:10.34133/research.0762)
Supplement: Supplementary 1 — Figs. S1 and S2 Tables S1 to S4 Data S1 [file research.0762.f1.zip › Supplementary Information.docx]

# Appendix A. Supplementary data


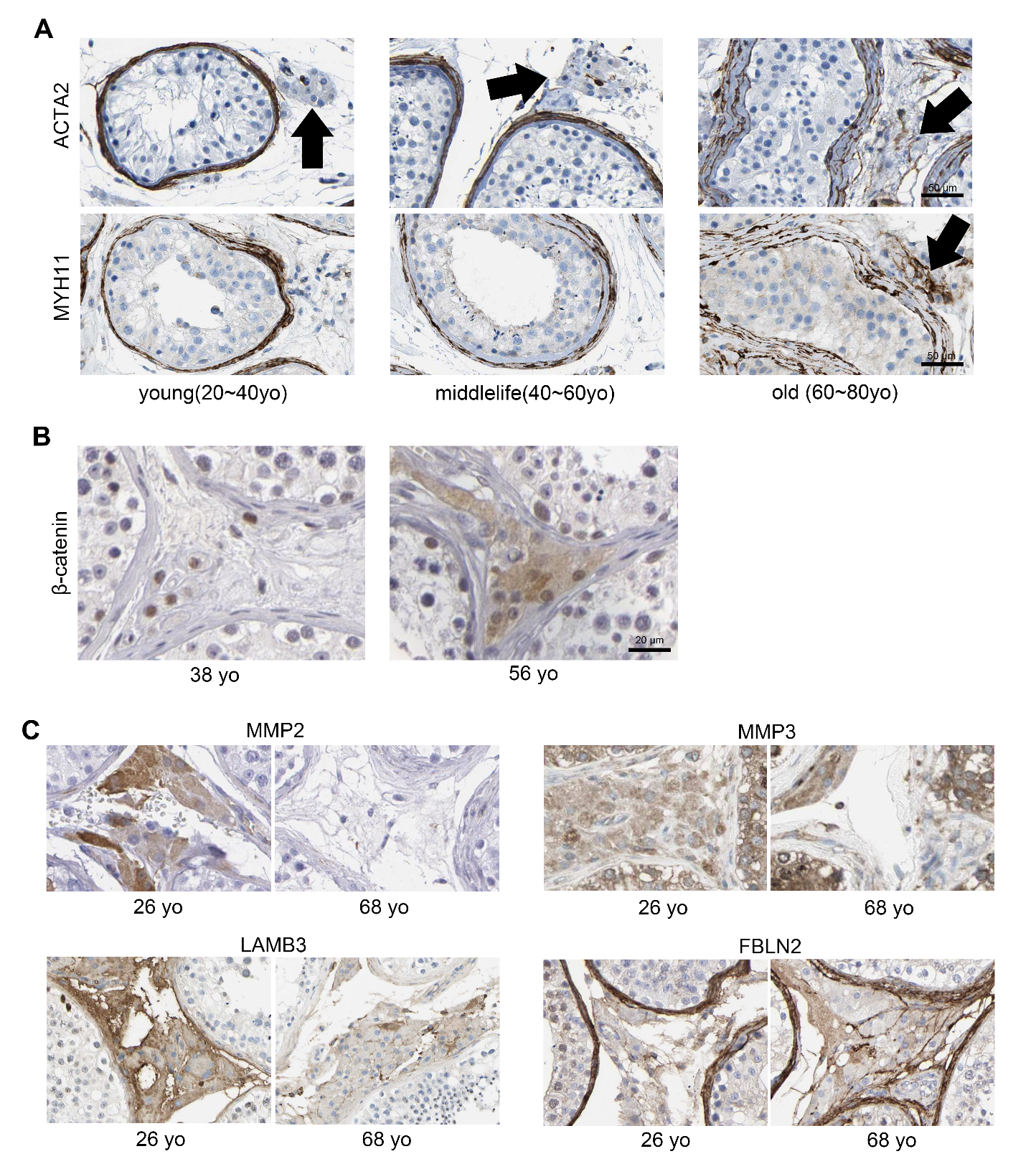


## Figure S1. The aging testicular interstitium exhibits a fibrotic phenotype.

(A-C) Immunohistochemistry images from The Human Protein Atlas database (<https://www.proteinatlas.org/>) present the expression of fibrosis-related proteins in the interstitial region of human testicular tissue across different age groups.


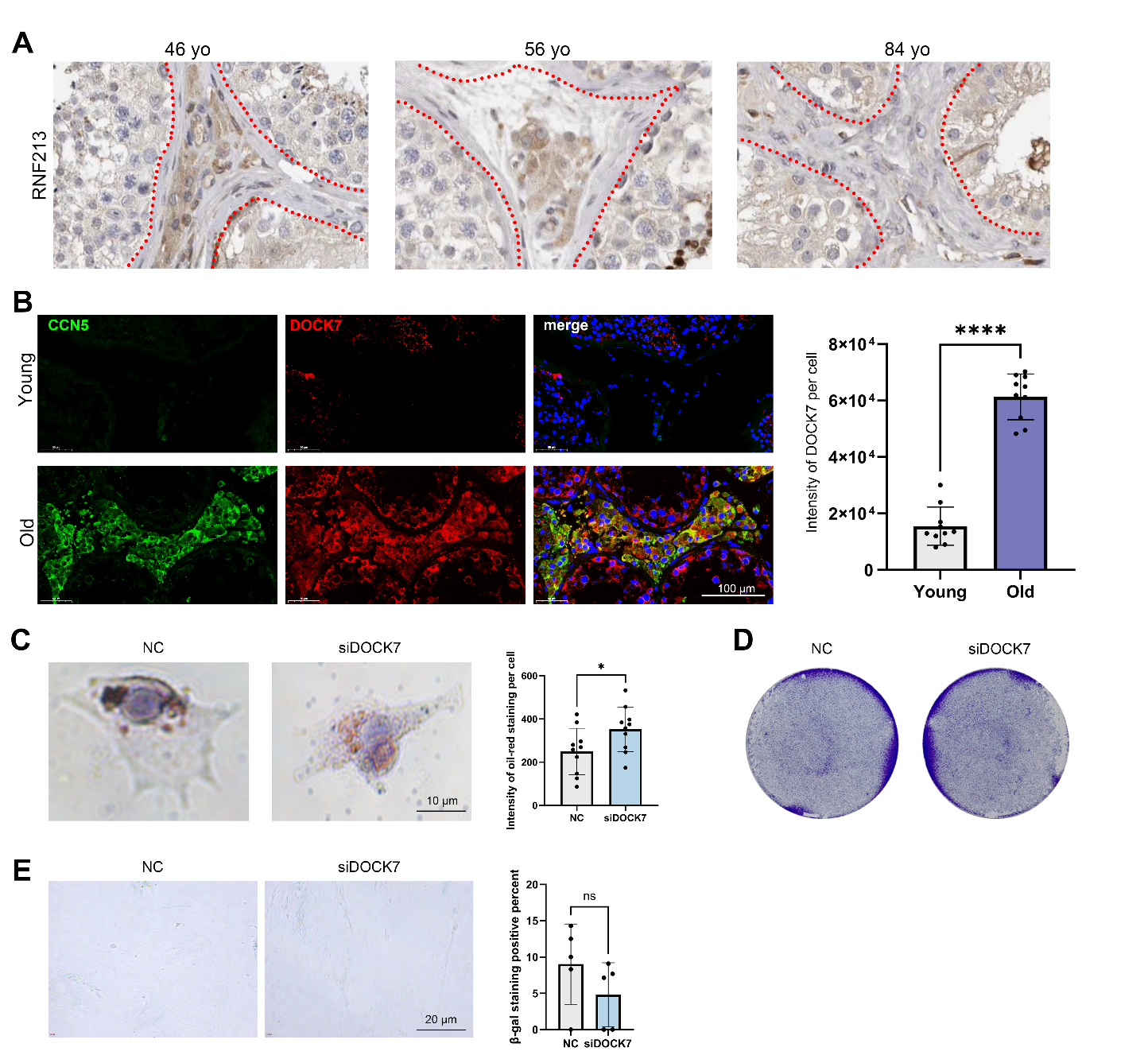


## Figure S2. DOCK7 affects lipid droplet metabolism in LCs.

(A) Immunohistochemistry images from The Human Protein Atlas database show the expression pattern of RNF213 in the interstitial region of human testicular tissue across different age groups.

(B) Immunofluorescence co-staining of DOCK7 (red) and CCN5 (green) in young and old mice testis. The scale bar represents 100 µm. n = 10 different regions.

(C) SA-β-gal staining of LCs with DOCK7 knockdown. The scale bar represents 20 µm. n = 5 technical repeats.

(D) Crystal violet staining marks colony formation in LCs with DOCK7 knockdown.

(E) Oil red staining of LCs with DOCK7 knockdown. The scale bar represents 10 µm. n = 10 cells.
